# Myelogenous Leukæmia and Its Treatment

**Published:** 1910-09-24

**Authors:** 

**Affiliations:** late Dean of the Faculty of Medicine, Member of the Academy of Medicine, Physician to the Beaujon Hospital, Paris.


### Hospital Clinics.

### MYELOGENOUS LEUKÆMIA AND ITS TREATMENT.

Clinical Notes on a Lecture by PROFESSOR DEBOVE, late Dean of the Faculty of Medicine, Member of the Academy of Medicine, Physician to the Beaujon Hospital, Paris.

Gentlemen,—As I have told you on more than one occasion, we lecturers have a marked tendency, in our clinics, to speak of the rather exceptional and out-of-the-ordinary cases. This is due to the fact that we are afraid of having to wait a long time before seeing such cases again in our wards. There is in this less inconvenience than you may imagine, for these patients give rise to very interesting problems of general pathology. We shall study to-day a case of myelogenous leukæmia.

### A Typical Case.

Mrs. D., æt. 32, occupation "posticheuse"—that is, she makes those mops of hair which you may observe on the heads of most women who have any pretension of being *a la mode*. Her parents died at the age of forty-one and forty-six respectively, but she cannot give any information as regards the cause of death. Two sisters dead, one in childbirth and the other at fifteen; she has four brothers living, all in good health. I may say here that up to the present no hereditary influence has been noted in the development of leukæmia, nor do infectious diseases appear to have any influence. Our patient has always enjoyed good health, except for typhoid at the age of twenty-seven. She is married since last year and has never been pregnant. She tells us that her malady began about a year ago; she lost strength, and her abdomen increased in volume to such an extent that she experienced difficulty in putting on her corset. Three months ago she had an attack of influenza, which greatly increased her state of weakness. She coughed a good deal at that time, and a physician whom she consulted diagnosed pulmonary tuberculosis; moreover, seeing her abdomen increased in size, he further stated that she was probably suffering from tuberculous peritonitis. Another medical man diagnosed ascites, whilst her friends suggested that she was pregnant. She then decided to come to the hospital for advice, and I may state at once that it was simply necessary to put the hand on her abdomen to discover that she had an enormous spleen.

### General Notes on the Case.

There is nothing special to note in her general appearance except that her abdomen is large; her general state of health seems fairly satisfactory. Simple inspection of the abdomen shows that it is not a case of pregnancy, for there is no increase of size in the lower and median part of that region of the body; it is the upper, and especially the left hypochondrium, which is abnormally developed. This, of course, immediately causes one to think of the possibility of a splenic tumour. On palpation, one finds that the right hypochondrium may be depressed quite freely, but that the left hypochondrium is distended by a voluminous, hard, and resistant mass which extends beyond the middle line and descends as low as the pubic arch. One can follow for some distance the right border of the spleen, and find it hard and fairly well marked. Percussion corroborates the information furnished by palpation. Examination of the liver shows that there is no increase in size of that organ. All the other viscera seem to be healthy; there are no pulmonary symptoms, no cough, no expectoration, and it is difficult to understand why the diagnosis of tuberculosis was made some little time ago. The heart presents a slight murmur of anæmic nature; the pulse is normal. There are no digestive symptoms worth noting; the urine is rather scanty, but contains neither sugar nor albumin. The only nervous trouble is rather marked asthenia. Contrary to what is generally observed in leukæmia, there has been no hæmorrhage and no adenitis in this case.

In fine, we may say that our patient has a large spleen, or, to be more exact, an enormous spleen, and a certain degree of anæmia and asthenia.

### The Blood in Leukæmia.

The splenomegalia naturally induced us to examine the blood, and this demonstrated the existence of leukæmia, and, what is more, of myelogenous leukæmia. Formerly one was quite satisfied with the fact that the blood contained red corpuscles and white corpuscles; if the latter were slightly increased in number the term leukocytosis was applied, and if their number was very greatly increased the state was called leukocythæmia.

To-day more careful study has shown that the white corpuscles are of different sorts, and that a lymphatic and a myeloid leukæmia must be distinguished. Lymphatic leukæmia is characterised by an increase of the number of lymphocytes, more or less similar to those existing in the normal blood. In myeloid leukæmia, besides normal lymphocytes there are also myelocytes—that is, mononuclear cells, the protoplasma of which contains granulations such as are found in the bone marrow. In other words, when the lymphocytes are in a state of hypergenesis, we are dealing with lymphatic leukæmia; when myelocytes exist in large numbers in the blood, we have myelogenous leukæmia.

### Microscopical Findings.

Having made these general remarks, we shall now proceed to consider the results of the blood examination in our patient; and as the characters of the normal blood may not be present in your minds I will recall them to you, and at the same time show in what measure our patient differs from the normal type.

*Red Corpuscles.*—In the normal state they number 5,000,000 per cubic millimetre; in our patient we find only 2,000,000. She presents, therefore, a state of anæmia. Normally there are no nucleated red cells in the blood; in our patient we have found some.

*White Corpuscles.*—The lymphocytes and the non-granular mononuclears which are found in the normal blood exist also in our patient, but, as is generally the case, they are markedly diminished in number.

The polynuclear elements do not present here any great divergence from the normal type:—

| Polynuclears. | Normal | the Patient. |
|---|---|---|
| Neutrophile. | 59 per cent. | 63 per cent. |
| Eosinophile. | 1 per cent. | 3 per cent. |

There is often a different state of matters, for it is frequent to note in these cases a notable increase of eosinophiles. The cells called mastzellen, which exist in small number in the normal blood, are not very numerous in the patient; in many cases of myelogenous leukæmia they are found greatly increased in number, and this has been considered an important feature of the disease. The myelocytes or granular mononuclears constitute the pathognomonic element of the affection; they do not exist in the normal state. In our patient they formed 24 per cent. of the white corpuscles. Myelocytes are divided according to their reaction to stains in neutrophile, eosinophile, and cells of the mastzellen type (differing from the real mastzellen in that their nucleus is round instead of polymorphous).

We may observe that several myelocytes are in a state of karyokinesis, which proves that they are diving; this is also proved by the fact that their protoplasma is mobile and presents pseudopodes. The sole presence of myelocytes allows us to state that we are dealing here with a case of myelogenous leukæmia. In this variety of the disease the serum often presents an opalescent tint, but this may exist in several other diseases, particularly in chronic nephritis. One may also often observe the presence of special crystals in the serum, the so-called "Charcot's crystals," the chemical composition and the diagnostic value of which are not perfectly known.

### Prognosis.

Let us now consider very briefly in what respects our patient differs from the ordinary clinical type generally observed. She has no enlargement of the glands, but this symptom is not a constant one. She has not up to the present had any hæmorrhage, although the latter is the rule in this disease, coming on in the form of epistaxis, bleeding from the gums, intestinal hæmorrhage, internal hæmorrhage, purpura, etc.

Allow me to remind you of the clinical characteristics which distinguish myelogenous from lymphatic leukæmia. The latter may present an acute form, recalling the acute infectious diseases, which may determine a fatal termination in a space of time varying from a few days to a few weeks. No acute form is observed in myelogenous leukæmia.

In lymphatic leukæmia the glands are always more or less affected, especially those of the neck and axillæ, whence the name of glandular leukæmia; in our patient the glands seem to be quite normal. In both forms the spleen is increased in size, but this is much more marked in the myelogenous variety. What especially distinguishes the two forms, however, is the blood formula.

I do not think that our patient will get well again, for all the cases that I have known or which have been followed up long enough have ended fatally in a space of time varying from six months to four years. Lymphatic leukæmia has a shorter course than the myelogenous form of the disease.

### Causation.

Leukæmia has been compared to cancer, but our information regarding the real nature of cancer is so vague that the comparison is relatively easy and does not advance us very much. Leukæmia seems to be due to an alteration in the hæmatopoietic organs, and as the latter are affected simultaneously and to the same degree in different parts of the body the course of the disease differs very much from that of cancer.

Is it due to the presence of a parasite? Many authorities think so; several have even described one. The existence of parasites, however, has not been confirmed by subsequent observation. It is quite possible that leukæmia is parasitic; at any rate, I would not advise anyone to make the experiment and have leukæmic blood injected into their veins. You may say that the experiment might be made on animals; it has often been tried, but without giving any results. Negative results, however, do not prove anything, for many diseases are not transmissible from one species to another, and I do not know of any case of leukæmia having developed in animals.

### Treatment.

Many kinds of treatment have been tried against this disease. I will mention some of them, for a certain degree of improvement may be obtained when they are properly applied. I will begin by eliminating surgical treatment. The spleen has been removed in patients suffering from leukæmia, but death has supervened in a short lapse of time after the operation. This might have been foreseen, for leukæmia is not simply a disease of the spleen, but affects also the bone marrow, and up to the present there has not been a surgeon bold enough to propose the removal of the latter.

Amongst the numerous drugs tried the most important are phosphorus, iodine, and quinine, and these seem in a certain number of cases to have improved the general state of health of the patients; but their action has not been so evident as to prove their usefulness against the disease itself.

Arsenic alone seems to have an influence on the disease and to produce a real improvement. It is prescribed in the form of Fowler's or Pearson's solution, but in these last few years it has been used especially in the form of cacodylate of soda, which can easily be injected under the skin and can be employed in doses which in the case of the other preparations of arsenic would give rise to toxic symptoms. For some time past, as you may know, leukolytic serums have been obtained—that is, serums which when introduced in the blood destroy the white corpuscles. This is a subject of great interest but too complex for me to enlarge upon here, and, besides, these serums have not given any results in the treatment of leukæmia.

Treatment by Animal Extracts.

Shall I speak of opotherapic treatment? Struck by the extraordinary success obtained in myxœdema by the use of thyroid extract, some physicians have surmised that the different tissues might have an action in the treatment of diseases, whence the use of diverse extracts of organs: of kidney in the diseases of the kidneys, of lung in the diseases of the lungs, and, in the case of leukæmia, of extracts of spleen, glands, and bone marrow. Up to the present this method has not produced any results; it is harmless, however, and if you or your patients so desire, there is no reason why you should not try it.

I come now to a relatively recent method of treatment of leukæmia—$x$-rays. Before describing their action in leukæmia I will just mention their action in healthy men and animals. When an animal is exposed to the influence of $x$-rays the constitution of the blood is modified, the lymphocytes undergo disintegration; the process may even go to practically the complete destruction of the white corpuscles. The lymphatic elements are also destroyed in the glands, the spleen, the bone marrow, the intestinal follicles; and it is not only the cellular elements which are destroyed, but the hæmatopoietic glands themselves undergo atrophy. Even after a quarter of an hour's exposure the effects of the rays are very appreciable.

Applied to the treatment of leukæmia $x$-rays have produced the following results: the leucocytes and myelocytes diminish in number; the spleen and the glands diminish in volume; a considerable quantity of uric acid is eliminated, probably due to the destruction of the white corpuscles; the body weight increases; the appetite improves; sleep and strength return.

But, you will say, this is splendid! Yes and No. Yes, because these results are most interesting in a disease which up to the present has resisted all the therapeutic means employed against it.

No, because I do not know of a single case in which definite recovery has been noted. All the patients observed for a sufficient length of time have relapsed and died.

